# Altered Insulin Receptor Substrate 1 Phosphorylation in Blood Neuron-Derived Extracellular Vesicles From Patients With Parkinson’s Disease

**DOI:** 10.3389/fcell.2020.564641

**Published:** 2020-12-03

**Authors:** Szu-Yi Chou, Lung Chan, Chen-Chih Chung, Jing-Yuan Chiu, Yi-Chen Hsieh, Chien-Tai Hong

**Affiliations:** ^1^Graduate Institute of Neural Regenerative Medicine, College of Medical Science and Technology, Taipei Medical University, Taipei, Taiwan; ^2^Ph.D. Program for Neural Regenerative Medicine, College of Medical Science and Technology, Taipei Medical University and National Health Research Institutes, Taipei, Taiwan; ^3^Department of Neurology, Shuang Ho Hospital, Taipei Medical University, New Taipei City, Taiwan; ^4^Department of Neurology, School of Medicine, College of Medicine, Taipei Medical University, Taipei, Taiwan; ^5^Graduate Institute of Biomedical Informatics, Taipei Medical University, Taipei, Taiwan; ^6^Master Program in Applied Molecular Epidemiology, College of Public Health, Taipei Medical University, Taipei, Taiwan

**Keywords:** Parkinson’s disease, insulin receptor substrate-1, biomarker, diabetes, extracellular vesicle

## Abstract

**Introduction:**

Diabetes increases the risk of Parkinson’s disease (PD). The phosphorylation of type 1 insulin receptor substrate (IRS-1) determines the function of insulin signaling pathway. Extracellular vesicles (EVs) are emerging as biomarkers of human diseases. The present study investigated whether PD patients exert altered phosphorylation IRS-1 (p-IRS-1) inside the blood neuron-derived extracellular vesicles (NDEVs).

**Research Design and Methods:**

In total, there were 94 patients with PD and 63 healthy controls recruited and their clinical manifestations were evaluated. Blood NDEVs were isolated using the immunoprecipitation method, and Western blot analysis was conducted to assess total IRS-1, p-IRS-1, and downstream substrates level in blood NDEVs. Statistical analysis was performed using SPSS 19.0, and *p* < 0.05 was considered significant.

**Results:**

The isolated blood EVs were validated according to the presence of CD63 and HSP70, nanoparticle tracking analysis and transmission electron microscopy. NDEVs were positive with neuronal markers. PD patients exerted significantly higher level of p-IRS-1^S312^ in blood NDEVs than controls. In addition, the p-IRS-1^S312^ levels in blood NDEVs was positively associated with the severity of tremor in PD patients after adjusting of age, sex, hemoglobin A1c, and body mass index (BMI).

**Conclusion:**

PD patients exerted altered p-IRS-1^S312^ in the blood NDEVs, and also correlated with the severity of tremor. These findings suggested the association between dysfunctional insulin signaling pathway with PD. The role of altered p-IRS-1^S312^ in blood NDEVs as a segregating biomarker of PD required further cohort study to assess the association with the progression of PD.

## Introduction

Insulin and the insulin signaling pathway are involved in the regulation of numerous essential cellular growth and metabolism processes (Haeusler et al., 2018). Insulin resistance, that is, an impaired cellular response to insulin stimulation, is the main characteristic of type 2 diabetes and contributes to downstream diabetes-related cellular and organ damage (Yaribeygi et al., 2019). The brain, especially neurons, is one of the major targets of insulin and is particularly vulnerable to insulin resistance (Gray et al., 2014). Alzheimer disease (AD), the most common neurodegenerative disease, is speculated to be associated with neural insulin resistance because the major AD pathogeneses, such as neurofibrillary tangles, neuroinflammation, oxidative stress, and neurodegeneration, are all associated with neural insulin resistance (Arnold et al., 2018). Furthermore, several studies have suggested that diabetes increases the risk of or accelerates the progression of Parkinson’s disease (PD), (Hu et al., 2007; Cereda et al., 2011; Sun et al., 2012; Lu et al., 2014; Hong et al., 2020). Moreover, no biomarker evidence exists to confirm the association between PD and neural insulin resistance.

Detecting neural insulin resistance in humans is challenging. In the cell, insulin activates insulin receptor tyrosine kinase, which phosphorylates and recruits type 1 insulin receptor substrate (IRS-1) protein. The phosphorylated IRS-1 (p-IRS-1) affect the function of insulin signaling pathway through regulating the downstream substrates. The levels of p-IRS-1/total IRS-1, therefore, can be cellular markers of the function of insulin signaling pathway (Copps and White, 2012). However, this cell-based assessment requires tissue biopsy, which is not feasible for PD, a neurodegenerative disease with the involvement of central nervous system (CNS). Therefore, neuron-derived extracellular vesicles (NDEVs) in blood is an ideal alternative (Budnik et al., 2016). Extracellular vesicles (EVs), small cargo secreted from all mammalian cells, carry abundant proteins and nucleotides from original cells. Exosome, a type of EV of diameter 30–100 nm, has attracted the most research attention because of their potential applications as biomarkers and in treatment (Shah et al., 2018). The inner content of NDEVs may mimic the cytoplasm of their original neurons (Pegtel et al., 2014), and the lipid bilayer outer membrane of EVs ensures their long-term stability in the blood. Additionally, NDEVs can cross the blood–brain barrier, and therefore, NDEVs in the peripheral blood can be used to detect the conditions of CNS neurons (Mustapic et al., 2017; Yang et al., 2018). Through the identification of a specific surface protein, L1 Cell Adhesion Molecule (L1CAM), NDEVs can be isolated from the blood (Fiandaca et al., 2015) and served as the platform of novel biomarkers identification of the neurological disease (Goetzl et al., 2019). The pathognomonic proteins of AD, such as β-amyloid and tau, which accumulate in neurons, have also been found in blood NDEVs in a high proportion (Lee et al., 2019; Watson et al., 2019). Regarding PD, EVs may augment the transmission of disease pathology (Han et al., 2019) and exosomal α-synuclein could be a biomarker for the development and progression of disease (Niu et al., 2020).

In addition to providing information on the cellular level of disease-related pathognomonic proteins, blood NDEVs can reveal the cellular functional status of neurons, such as the function of insulin signaling pathway through the status of p-IRS-1/IRS-1 (Kapogiannis et al., 2015). Currently, the association between the functional of neuronal insulin signaling pathway and PD has to be clearly confirmed because diabetes may be one of the few modifiable risk factors for PD, and a few Food and Drug Administration-approved antiglycemic agents have demonstrated neuroprotective effects on PD experimental models (Dehmer et al., 2004; Quinn et al., 2008; Ismaiel et al., 2016; Katila et al., 2017). Unlike the known association between AD and dysfunctional p-IRS-1 in blood NDEVs (Mullins et al., 2017), the association between blood p-IRS-1/IRS-1 and PD is unknown. It had only been applied on a clinical trial to investigate the therapeutic response of exenatide on PD patients (Athauda et al., 2019). The present study hypothesizes that dysfunctional insulin signaling pathway is associated with PD and can be identified through altered p-IRS-1 in blood NDEVs. In addition, this phenomenon may correlate with PD severity.

## Materials and Methods

### Study Participants

In total, 157 participants (94 people with PD and 63 healthy controls) were enrolled in this study. PD diagnoses were based on the United Kingdom Parkinson’s Disease Society Brain Bank Diagnostic Criteria (Hughes et al., 1992). Only people with mild to moderate PD, defined as stage I–III PD according to the Hoehn and Yahr stage, were included. Healthy controls were free from known neurodegenerative, psychiatric, and major systemic diseases (malignant neoplasm and chronic kidney disease) and were regularly followed up in outpatient clinics for chronic conditions (hypertension, diabetes, or hyperlipidemia). This study was approved by the Joint Institutional Review Board of Taipei Medical University (approval nos. N201609017 and N201801043). The informed consent was obtained directly from the study participants in the outpatients clinic after the explanation of this study by the attending neurologists (CC, LC, and CH).

### Clinical Assessments

All participants were interviewed to obtain their baseline demographic data. The cognitive functions of all study participants were investigated by trained nurses using the Taiwanese versions of the Mini-mental state examination (MMSE) and Montreal cognitive assessment (MoCA). All PD participants were evaluated using Parts I, II, and III of the unified Parkinson’s disease rating scale (UPDRS) during an outpatient visit. The time between the most recent dose of anti-PD medication and the assessment of UPDRS Part III was not recorded, and patients with PD were assumed to be on time. The subgrouping of the UPDRS Part III into the categories of tremor, akinetic rigidity and postural instability and gait disturbance (PIGD) subtype was modified according to the previous literature (Lewis et al., 2005). In brief, tremor score was from the subitem 20 and 21 of UPDRS; PIGD from the subitem 27, 29, and 30; akinetic rigidity from subitem of 18, 22, 23, 24, 25, 26, and 31.

### Blood NDEVs Isolation and Validation

The blood sampling was performed through venipuncture during the outpatient clinic visit, at which fasting was not requited. The process blood sample, isolation of plasma and the storage were followed the recommendations from International Society for Extracellular Vesicles (Théry et al., 2018). Plasma samples from both healthy controls and PD patients were purified for EVs isolation by using an ExoQuick Plasma Prep with Thrombin kit (System Biosciences, EXIQ5TM-1, Palo Alto, CA, United States) according to the manufacturer’s protocol. In brief, 250 μL plasma was mixed with 2.5 μL thrombin and rotated at room temperature for 5 min. Then, it was centrifuged at 12,000 rpm for 5 min at 4°C. In a new test tube, 200 μL supernatant was mixed with 50 μL ExoQuick solution at 4°C for 1 h followed by centrifugation at 1,000 *g* for 30 min. After the supernatant was removed, EVs pellets were further immunoprecipitated with 1 μg anti-L1CAM antibody (clone UJ127.11, Sigma-Aldrich) and Dynabeads^TM^ protein A beads with DynaMag^TM^-2 system (Thermo Fisher) for NDEVs isolation. The mixtures were incubated on the rotator (Multi Bio RS-24, Biosan) in 4°C for 12 h with 11 rpm speed and 1 s orbital rotation. The turning angle and time of reciprocal motion was 90° and 3 s. The turning angle of vibro motion was 5° and 5 s. Plasma EVs from randomly selected six health controls and PD patients were diluted with exosome-grade PBS (Cat.17-516L, Lonza) for nanoparticle analysis by using a Nanoparticle Tracking Analyzer (NTA, NanoSight NS300, Malvern Panalytical, Malvern, United Kingdom). The capture and analysis settings of the NTA were kept constant among all samples as following table.

**Table:** 

Capture settings	
Camera type	sCMOS
Laser type	Blue 488
Number of capture	3
Capture duration	60 s
Camera level	16
Slider shutter	1,300
Slider gain	512
FPS	25.0
Temperature	25.0°C
**Analysis settings**	
Detect threshold	5
Screen gain	10.0

### Transmission Electron Microscopy

Morphological analysis and imaging of EVs isolated from plasma were performed using a Hitachi 7700 transmission electron microscopy (TEM; Hitachi High-Tech Corporation. Tokyo, Japan). In brief, EVs were transferred onto a filmed grid (F077/N050, Aldermaston, Berks, United Kingdom) in chloroform immediately after isolation. The grids were then stained with 2% (v/v) uranyl acetate in double-distilled water just before examination.

### IRS-1 and p-IRS-1 Level Assessment and Antibodies Used in Study

Immunoprecipitated NDEVs were directly lysed using protein sample buffer (NuPAGE^TM^ LDS Sample Buffer, Thermo Fisher) and analyzed using protein sodium dodecyl sulfate and polyacrylamide gel. IRS phosphorylation status and exosome marker were detected using specific antibodies. Human brain lysate was purchased from GeneTex (GTX27918). Antibodies against IRS-1 Ser636/639 (#2388), total IRS-1 (#2382), Akt pan (#4691), Akt S473 (#4060), extracellular signal-regulated kinases (Erk) (#4695), phospho Erk (#4380), p70 S6 Kinase (49D7, #9203), Phospho-p70 S6 Kinase (Thr389, #9234), and Phospho-p70 S6 Kinase (Thr421, #9204) were purchased from cell signaling. Antibodies for IRS-1 T612 (44-816G), IRS-1 S616 (44-550G), and CD63 (10628D) were purchased from Thermo Fisher. Antibodies for glyceraldehyde 3-phosphate dehydrogenase (GADPH) (GTX100118), GFAP (GTX1108711), COX4 (GTX114330) were from GeneTex. Tau-1 (MAB3420), L1CAM (ab24345), GluR1 (04-823), GluR2 (MAB397), and IRS-1 S312 (clone 24. 6.2, #05-1087) were purchased from Millipore, and heat shock proteins (HSP) 70 (NBP1-77456) was from NOVUS. L1CAM (L4543) used for immunoprecipitation which was from Sigma. NR2A (75-288) was from NeuroMab. Antibodies were prepared in tris-buffered saline containing 0.2% Tween 20 (TBST) and 3% BSA (Bovine serum albumin). Secondary antibodies including anti-Mouse IgG conjugated HRP (115-035-003) and anti-Rabbit IgG conjugated HRP (111-035-003) were purchased from Jackson ImmunoResearch. Protein blot intensities were quantified using Image J software. The expression level of phosphorylated protein was normalized to the total form in the same patient. The others were normalized to GADPH. For making sure the data can be comparable among gels, all of the data was normalized to the average of control group in the same gel.

### Statistical Analysis

All statistical analyses were performed using SPSS for Windows 10 (version 19; SPSS Inc., Chicago, IL, United States). Non-parametric Kolmogorov–Smirnov test was used to compare the continuous variables, including age, hemoglobin A1c, body mass index (BMI), scores of the clinical assessments and total IRS-1, p-IRS-1 and downstream substrates levels in blood NDEVs between PD patients and controls. Fisher’s Exact test was used to compare the categorical variables between PD patients and controls, such as sex and the presence of diabetes. Spearman’s rank test was used to investigate the correlation between p-IRS-1^S312^ and p-IRS-1^S616^ with the severity of motor symptoms in PD patients. Multivariable linear regression was used to adjust the confounding factors, including age, sex, HbA1c, and BMI. One-way ANOVA with Dunnett’s *post hoc* analysis was used for multiple comparison. Data was presented as mean ± standard deviation (SD) if no specific description. We considered *p* value of <0.05 as statistically significant.

## Results

### Blood NDEV Validation

**FIGURE 1:**
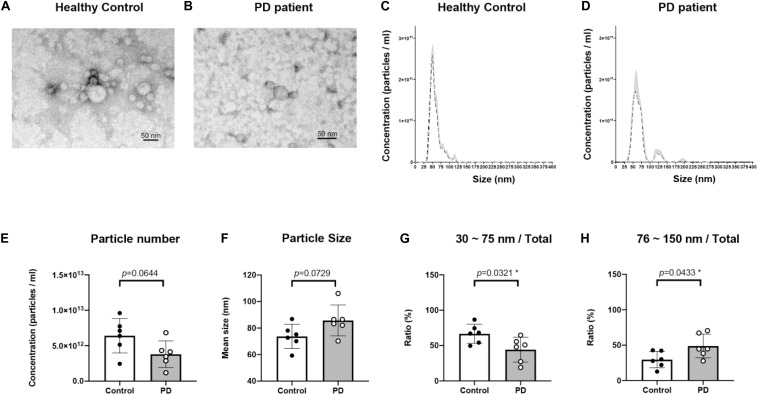
Characterization of blood neuron-derived extracellular vesicles from healthy controls and Parkinson’s disease (PD) patients. The particle size distribution of plasma extracellular vesicles from both healthy control and PD patients’ was analyzed by electron microscopy **(A,B)** and NTA (data are presented as mean ± SEM) **(C,D)**. The **(E)** concentration and **(F)** average size of EVs from healthy controls and PD patients were not different. The percentages of EVs of sizes **(G)** 30–75 nm and **(H)** 75–150 nm varied in PD patients *p < 0.05.

Plasma EVs were isolated using the ExoQuick Plasma kit (see section “Materials and Methods”). Isolated EV particles were validated using TEM (Figures 1A,B) and NTA (Figures 1C,D). The particle size was mainly distributed from 30 to 120 nm (Figures 1C,D). The concentration and the average size of EVs isolated from plasma were not different between healthy controls and PD patients (Figures 1E,F). However, EVs of sizes between 30 and 75 nm were more in healthy controls than in PD patients (*p* = 0.0321; Figure 1G); the majority of EVs isolated from PD patient plasma were 75–150 nm in size (*p* = 0.0433; Figure 1H). Thus, the EVs population in PD patient blood may vary.

### Demographic Data of the Study Participants

The baseline data are presented in Table 1. No differences were noted in age, sex, education level, or BMI between the PD patients and controls. PD patients exhibited significantly lower rate of diabetes (PD, 14 out of 94; control, 21 out of 63; *p* = 0.01) and worse cognition (PD: 25.18 ± 5.24; control: 27.33 ± 2.79 which were assessed by MMSE with total score = 30; *p* = 0.03). PD patients exerted a trend of lower HbA1c (PD, 6.02 ± 0.93; control, 6.15 ± 1.18%; *p* = 0.06). For PD patients, the mean disease duration was 2.85 ± 2.47 years, and the mean score of UPDRS Part III was 22.77 ± 9.60. The anti-PD medications for the PD patients included levodopa (*n* = 83, median dosage = 300 mg/day), ropinirole (*n* = 34, media dosage = 4 mg/day), rotigotine (*n* = 13, median dosage = 6 mg/day), pramipexole (*n* = 11, median dosage = 0.75 mg/day), rasagiline (*n* = 10, median dosage = 0.5 mg/day), amantadine (*n* = 8, median dosage = 200 mg/day), and selegiline (*n* = 2, median dosage = 7.5 mg/day).

**TABLE 1:** Demographic data of the study participants.

	Control, *n* = 63	PD, *n* = 94	*P* value
Age, years	67.97 ± 7.57 (52∼89)	69.00 ± 8.20 (50∼99)	0.53
Female	24	46	0.15
MMSE	27.33 ± 2.79 (16∼30)	25.18 ± 5.24 (2∼30)	0.03
MoCA	23.22 ± 3.92 (11∼29)	20.91 ± 6.26 (0∼30)	0.06
Diabetes	21	14	0.01
HbA1c,%	6.15 ± 1.18 (5.00∼9.00)	6.02 ± 0.93 (4.3∼10.6)	0.06
BMI	25.38 ± 3.02 (19.72∼31.74)	25.39 ± 3.56 (17.96∼38.10)	1.00
Education > 12 years	9	22	0.16
Disease duration, years		2.85 ± 2.47 (0∼15)	
UPDRS-III		22.77 ± 9.60 (2∼52)	

*MMSE, mini-mental status examination; MoCA, Montreal Cognitive Assessment; HbA1c, glycated hemoglobin; BMI, body mass index; UPDRS, unified Parkinson’s disease rating scale; PD, Parkinson’s disease. Data was presented as mean ± SD (minimum∼maximum).*

### Blood NDEV IRS-1 and p-IRS-1 Levels in the PD Patients and Controls

The NDEVs was further purified with L1CAM immunoprecipitation. The neuronal proteins—MAP-2, Tau-1, Synaptophysin and L1CAM were analyzed. Compared to NDEVs and brain lysate, total plasma EVs expressed lower level of MAP2 and Tau protein. Mitochondria protein—COX4 was found in brain lysate only but not NDEVs and total EVs (Supplementary Figure 1).

**FIGURE 2:**
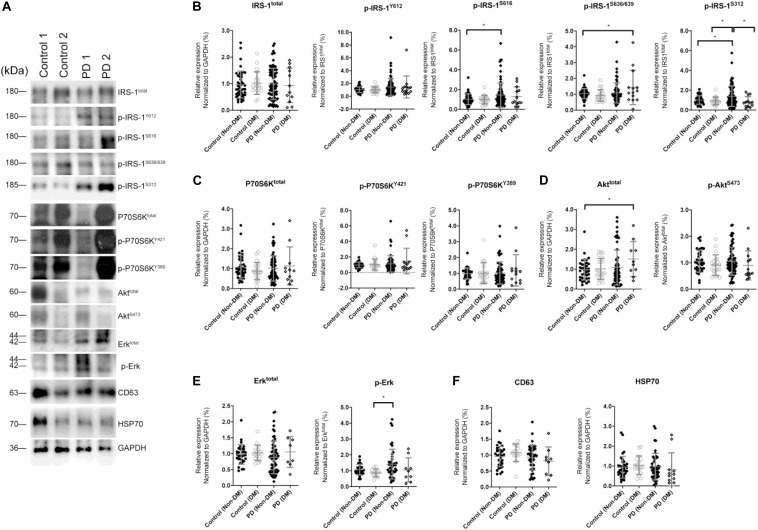
Total IRS-1, p-IRS-1 and downstream substrates level in blood neuron-derived extracellular vesicles. **(A)** The representative protein blot images of IRS-1 and different phosphorylated forms of IRS-1, including Y612, S616, S636/639, and S312. HSP70 and CD63 were exosomal proteins and markers. GAPDH was the protein loading control (1 and 2 indicate different samples). Comparison of the levels of total IRS-1, p-IRS-1^Y612^, p-IRS-1^S616^, p-IRS-1^S636/639^, p-IRS-1^S312^ **(B)**, and downstream IRS-1 substrates **(C–E)** between healthy controls [with or without diabetes mellitus (DM)] and Parkinson’s disease (PD) patients (with or without DM). The phosphorylation status of IRS-1, P70S6K, Akt, and Erk was normalized to total form IRS-1, P70S6K, Akt, and Erk. **(F)** The EV markers–CD63 and HSP70 was not different between 4 groups. Data was presented as mean ± SEM. *p < 0.05.

The levels of total IRS-1 and tyrosine/serine p-IRS-1 in blood NDEVs were analyzed in PD patients and control (Figure 2A as representative figure and summarized in Supplementary Figure 2). HSP70 and CD63 are EVs markers. There was no significant difference regarding the total IRS-1 level in PD patients compared with controls. Significant elevation of the level of p-IRS-1^Y612^, p-IRS-1^S616^, and p-IRS-1^S312^ were noted in PD patients. In the downstream substrates of IRS-1, phosphorylated Erk (Figure 2E and Supplementary Figure 1D) was significantly increased in the PD patients whereas no significant difference was found from the rest of substrates such as Akt and P70S6 Kinase (Figures 2C,D and Supplementary Figures 2B,C). However, considering diabetes may affect the p-IRS-1 in the neurons, people with diabetes in both control and PD group were separated into individual subgroup. Significant elevation of p-IRS-1 in non-diabetes PD patients compared with non-diabetes control was only observed in the p-IRS-1^S312^ and p-IRS-1^S616^ (Figure 2B). If further taking out the impaired cognition controls (MMSE < 27), p-IRS-1^S312^ remained significantly elevated in PD patients compared with normal cognition controls (Supplementary Table 1). In addition, there was no significant difference between the non-diabetes PD patients and controls in the downstream substrates level in blood NDEVs (Figures 2C–E). For PD patients, the level of p-IRS-1^S312^ in blood NDEV was significantly elevated compared with the normal cognition controls after adjusted the effect of age, sex, HbA1c, and BMI (Table 2). The EVs level was not different among four groups regarding to CD63 and HSP70 levels (Figure 2F and Supplementary Figure 2E).

**TABLE 2:** The association of Parkinson’s disease with the neural-derived exosomes total and phosphorylated IRS-1 level between PD patients with normal cognition controls (mini-mental state test>26) after the adjustment of age, sex, HbA1c, and body mass index among people without the diagnosis of diabetes.

	Standardized coefficient (β)	95% confidential interval	*p* value
IRS-1^total^	−0.065	−0.234∼0.129	0.569
p-IRS-1^Y 612^	0.168	−0.018∼0.144	0.126
p-IRS-1^S 616^	0.193	−0.009∼0.164	0.080
p-IRS-1^S 636/639^	0.163	−0.166∼0.196	0.871
p-IRS-1^S 312^	0.235	0.009∼0.224	0.035*

* p < 0.05.

### p-IRS-1^S312^ in Blood NDEVs Was Associated With the Severity of Tremor in PD Patients

**FIGURE 3:**
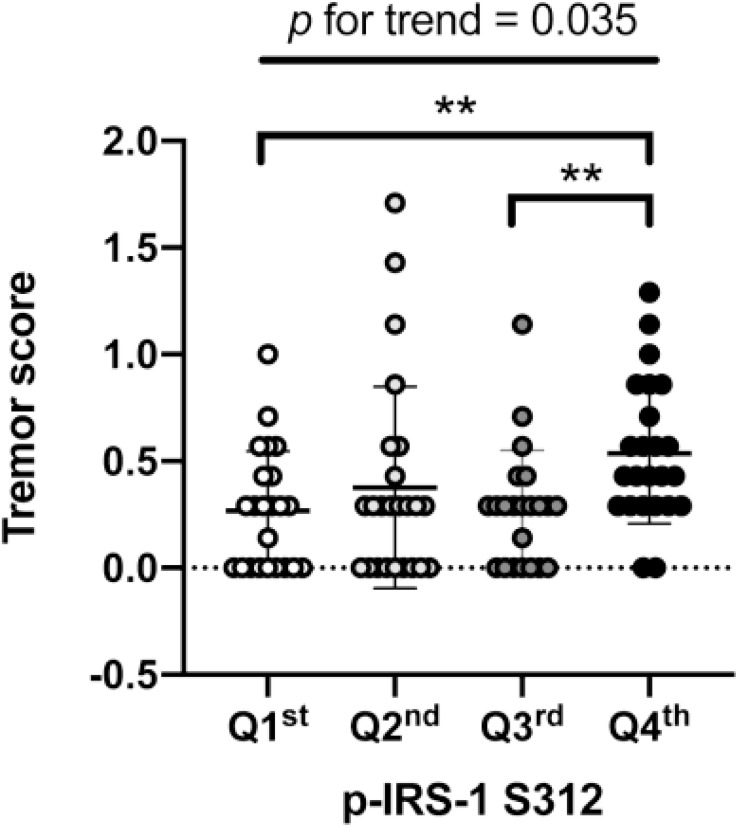
Association of p-IRS-1^S312^ with the severity of tremor in Parkinson’s disease (PD) patients. The box plot demonstrated the severity of tremor in PD patients with different amount of p-IRS-1^S312^ in blood NDEVs. Subgrouping of PD patients based on the quartile of p-IRS-1^S312^ level in blood NDEs. The box plot presented the data as median, first quartile and third quartile with the whiskers presented the minimum and maximum. Outliers were presented as hallow circle. **p < 0.01.

In PD patients, the level of p-IRS-1^S312^ but not p-IRS-1^S616^ in blood NDEVs were significantly associated with the severity of resting and action tremor (Supplementary Table 2). The level of p-IRS-1^S312^ and p-IRS-1^S616^ were both not associated with the disease duration (Supplementary Figure 2). Further categorizing the motor symptoms into three aspects: tremor, akinetic rigidity and PIGD, only tremor was significantly associated with the level of p-IRS-1^S312^ but not p-IRS-1^S616^ in blood NDEVs after the adjustment of age, sex, BMI, and HbA1c (Table 3). Further subgrouping of PD patients based on the quartile of the level p-IRS-1^S312^ in blood NDEVs, it was found that the severity of tremor was significantly different between the four subgroups of PD patients (*p* for trend = 0.035) (Figure 3). The *post hoc* analysis also demonstrated that the significant difference was noted between Q1–Q4 (*p* = 0.028), and Q3–Q4 (*p* = 0.035).

**TABLE 3:** The association between pIRS-1^S312^ and pIRS-1^S616^ with the three major categorial motor symptoms of PD after the adjustment of age, sex, HbA1c, and body mass index in all PD patients.

	p-IRS-1^S312^		p-IRS-1^S616^	
	Standardized coefficient (β)	*p* value	Standardized coefficient (β)	*p* value
Tremor	0.235	0.047*	0.060	0.618
Akinetic rigidity	−0.112	0.349	−0.061	0.613
Postural instability and gait disturbance	−0.126	0.264	−0.047	0.680

* p < 0.05.

In PD patients, p-IRS-1^S312^ was negatively associated with total IRS-1 level, and positively associated with p-IRS-1^Y612^ and p-IRS-1^S616^. Regarding the downstream substrates of IRS-1, p-IRS-1^S312^ was not significantly associated with any selected targets (Supplementary Table 3). None of the downstream substrates was associated with the severity of any clinical item significantly (Supplementary Table 4).

## Discussion

The present study demonstrated that in blood NDEVs, the p-IRS-1^S312^ level was significantly increased in either overall and non-diabetes PD patients compared with their counterpart controls. Additionally, the p-IRS-1^S312^ level in blood NDEVs significantly correlated with the severity of tremor after the adjustment of age, sex, BMI, and HbA1c, which indicated the possible role of altered p-IRS-1^S312^ as a disease segregating biomarker. Although the levels of p-IRS-1^S312^ certainly overlapped between controls and PD patients, to the best of our knowledge, the present study is the first to provide biomarker-based evidence for the association between the altered neuronal p-IRS-1 with PD by means of the analysis of blood NDEVs, and this observation also hinted the role of dysfunctional insulin signaling pathway in the pathogenesis of PD.

Type 2 diabetes exerts wide-spectrum detrimental effects on cells, tissues, and organs (Mauricio et al., 2020). A growing body of evidence has demonstrated an increased PD risk for patients with diabetes. In addition, diabetes and poor blood sugar control are linked to fast deterioration and worse PD severity (Cereda et al., 2012; Kotagal et al., 2013). Although insulin resistance or diabetes enhances PD-related pathogeneses in *in vivo* and *in vitro* neuronal models (Bousquet et al., 2012; Solmaz et al., 2017; Hong et al., 2020), biological evidence in human studies is lacking and the functional assessment of insulin signaling pathway in PD patients’ neurons is challenging. The fasting or postprandial blood glucose and HbA1c test reflect the pancreatic islet function but not insulin resistance (Desimone and Weinstock, 2000). The altered p-IRS-1 can be an indicator of dysfunctional insulin signaling pathway, which is feasible in skeletal muscles or adipocytes but not in CNS due to difficulty in obtaining the neuronal tissue (Le Roith and Zick, 2001; Zick, 2001). The present study investigated the p-IRS-1 and the downstream substrates in blood NDEVs, which achieved two goals simultaneously. Increased p-IRS-1^S312^ and p-IRS-1^S616^ in blood NDEVs indicated the dysfunctional insulin signaling pathway in PD patients, which supports the association reported in previous epidemiological studies about diabetes and PD.

Type 1 insulin receptor substrate has numerous phosphorylation sites, which are responsible for different functions individually. Ser616, the most studied phosphorylation site, is located exactly at the phosphoinositide 3-kinase (PI3K) binding domain, which promotes the downstream protein kinase B pathway and subsequent cell metabolism. Conversely, insulin activates other signaling pathways and kinases, and their negative feedback induces the Ser312 phosphorylation of IRS-1 and inhibits its functions (Gual et al., 2005). Furthermore, p-IRS-1^S312^ triggers IRS-1 degradation through the PI3K pathway (Greene et al., 2003). However, it also had been found that in mice, p-IRS-1^S307^ (human S312) positively regulated the severity of insulin resistance by maintaining proximal insulin signaling (Copps et al., 2010). These conflicts may indicate the existence of a well-regulated feedback loop for the phosphorylation of IRS-1, and the possible dual roles of each phosphorylation site in the regulating of insulin signaling pathway. Clinically, on the hippocampal formation of people with AD, p-IRS-1^S616^ increase was associated with a reduction of insulin response, the oligomeric of Aβ plaques, and the memory (Talbot et al., 2012). The present study demonstrated that in PD patients, the p-IRS-1^S312^ and p-IRS-1^S616^ level in blood NDEs increased compared with non-PD controls, but only p-IRS-1^S312^ was associated with the severity of tremor, one of the most remarkable motor symptoms of PD. Increased p-IRS-1^S312^ but not p-IRS-1^S616^ expression levels was noted in PD patients in the post-mortem study and mutant α-synuclein over-expressing animal model (Bassil et al., 2017). This discrepancy may require further *in vitro* and *in vivo* studies to delineate the clear causal relationship.

The strength of the present study is that it provides the cell biology-based association between PD and altered neuronal IRS-1 phosphorylation. Blood NDEVs, which had promising roles in the study of PD [reviewed by Porro et al. (2019) and Yu et al. (2020)] provide neuronal information directly without the interference from peripheral. The increase in p-IRS-1^S312^ in PD blood NDEVs may hint dysfunctional insulin signaling pathway in the neurons of PD patients. Although the causal relationship between insulin resistance and PD is not well defined, numerous antiglycemic agents exhibit possible disease modification effects on PD. The results of the present study may facilitate the development of novel treatment because of a better understanding of the relationship, and the p-IRS-1 in the neurons may also serve as a biomarker of the therapeutic effect of antiglycemic agents in clinical trials [Athauda et al., 2017; Ninds Exploratory Trials in Parkinson Disease (Net-Pd) Fs-Zone Investigators, 2015]. In fact. the total/phosphorylated IRS-1 and the downstream substrates had been investigated in a phase II clinical trial, which investigated the disease modification effect of exenatide (Athauda et al., 2019). In that study, both tyrosine and serine p-IRS-1 were elevated in the treatment group of PD patients, and the downstream substrates elevation was associated with the improvement of motor symptoms. Compared with that study which enrolled PD patients only, the present study made the comparison between PD patients and controls. Moreover, the increase in the p-IRS-1^S312^ level in blood NDEVs was associated with greater severity of tremor. Tremor dominant PD is distinct form other subtypes (akinetic rigidity and PIGD) of PD in the clinical presentations, comorbidity and the brain pathology (von Coelln and Shulman, 2016). Cerebellum is known to be the origin of the tremor (Wu and Hallett, 2013) and diabetes induces the PD pathology in the cerebellar Purkinje cells *in vivo* (Solmaz et al., 2017), which may be responsible for the association between altered p-IRS-1 in blood NDEVs with the severity of tremor. This relationship suggested p-IRS-1^S312^ in blood NDEVs as a potential segregating biomarker of PD, although a follow-up study is warranted to confirm the prediction accuracy. Lastly, obtaining blood NDEVs was more convenient than obtaining CSF, and the stability of exosomes makes long-term storage and long-haul transport possible, which facilitates further large-scale international PD cohort studies.

The present study provided some interesting insights but also had some limitations. It is a single-centered study, which required further study for validation in different institutes, races and countries. The control subjects were not the healthy controls like other study but relative a disease-control. They were age, sex matched population who regularly visit outpatient clinic for controlling conventional vascular risk factors (hypertension, diabetes, and hyperlipidemia). Some of them, although did not have subjective memory decline, may already fell into the category of mild cognitive impairment or mild dementia, which indicated the comorbidity with vascular or AD pathology in the control group. However, this approach was able to generate a control with similar cognitive ability with PD patients in the study, and obtained the PD-specific but not overall neurodegeneration-related neuronal insulin resistance comparison. Second limitation was the lack of association between the downstream substrates of insulin signaling pathway with either the p-IRS-1 or the clinical presentations. Nevertheless, those proteins, such as Akt and Erk, were regulated by multiple molecular pathways. The other pathogeneses of PD, such as mitochondrial dysfunction, neural inflammation and lysosomal dysfunction, may also affect these substrates, and those substrates may not be packed into EVs. In addition, several serine phosphorylation sites are available on IRS-1, and the present study demonstrated that while p-IRS-1^S312^ was associated with PD, and the severity of tremor in PD patients. The underlying pathogenesis related to this specific phosphorylation must be addressed in basic *in vitro* and *in vivo* studies of PD, which may be beneficial for understanding disease pathogenesis and drug development. The overlap between PD patients and control in the level of p-IRS-1^S312^ in blood NDEVs may limit the application of p-IRS-1 as a diagnostic biomarker for PD, but the association with tremor may provide another role as the segregating biomarker for PD. Lastly, it was a cross-sectional study, which required further cohort study to validate the association between the role of p-IRS-1 in blood NDEVs with the progression of PD.

In conclusion, altered p-IRS-1 in blood NDEVs was observed in PD patients compared with controls. In addition, higher p-IRS-1^S312^ in blood NDEs was noted in PD patients with greater severity of tremor. These findings provide not only the basis of the previously reported epidemiological association between PD and diabetes but also the possibility of developing a novel blood biomarker of PD. We recommend further investigation of p-IRS-1 in blood NDEVs in PD patients longitudinally for examining its correlation with multiple aspects of disease progression.

## Data Availability Statement

The raw data supporting the conclusions of this article will be made available by the authors, after the permission of TMU-JIRB.

## Ethics Statement

The studies involving human participants were reviewed and approved by the Joint Institutional Review Board of Taipei Medical University (approval nos. N201609017 and N201801043). The patients/participants provided their written informed consent to participate in this study.

## Author Contributions

C-TH and S-YC: study design. C-TH, LC, and C-CC: patient recruitment. S-YC, J-YC, and Y-CH: blood sample analysis. S-YC, J-YC, Y-CH, and C-TH: data analysis. LC, C-CC, C-TH, and S-YC: manuscript drafting and revision. All authors read and approved the final manuscript.

## Conflict of Interest

The authors declare that the research was conducted in the absence of any commercial or financial relationships that could be construed as a potential conflict of interest.
