# Supplementary material for: Altered Insulin Receptor Substrate 1 Phosphorylation in Blood Neuron-Derived Extracellular Vesicles From Patients With Parkinson’s Disease
Source: Front Cell Dev Biol. 2020 Dec 3;8:564641. doi: 10.3389/fcell.2020.564641 (PMC7744811; doi:10.3389/fcell.2020.564641)
Supplement: Supplementary file 1 [file Data_Sheet_1.docx]

**Supplementary Information**

**Supplementary Figure 1:** The expression of neuronal and EVs related proteins in NDEVs, total plasma EVs and mouse brain lysate.

**
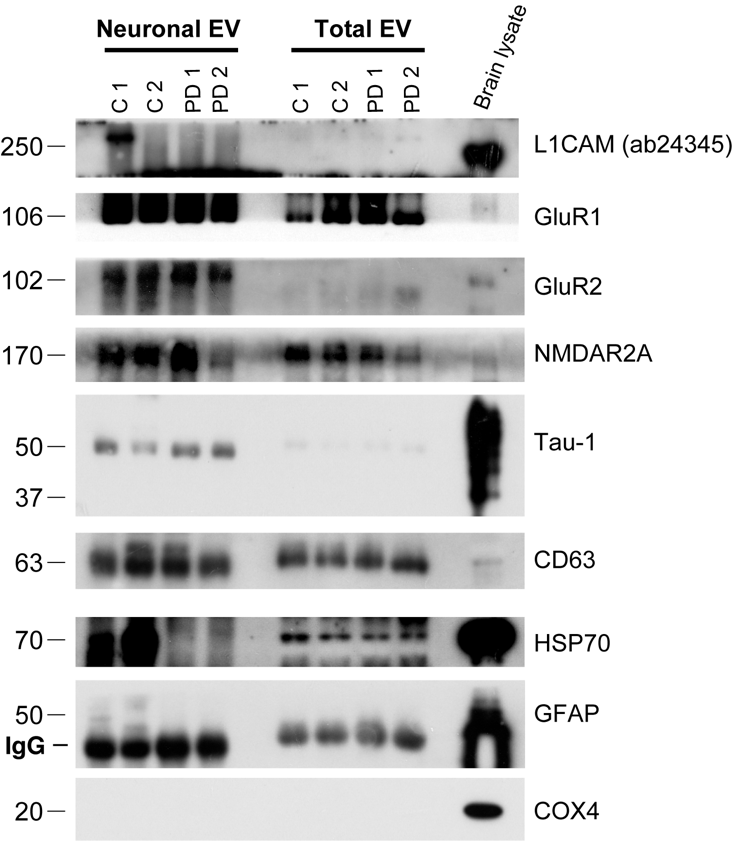
**

**Supplementary Figure 2:** The comparison of the level of IRS-1, p-IRS-1 (A) and downstream singling pathways substrates (B, C, D) from blood NDEVs in overall PD patients and control. (E) The EVs markers CD63 and HSP70 level in PD patients and control.

**
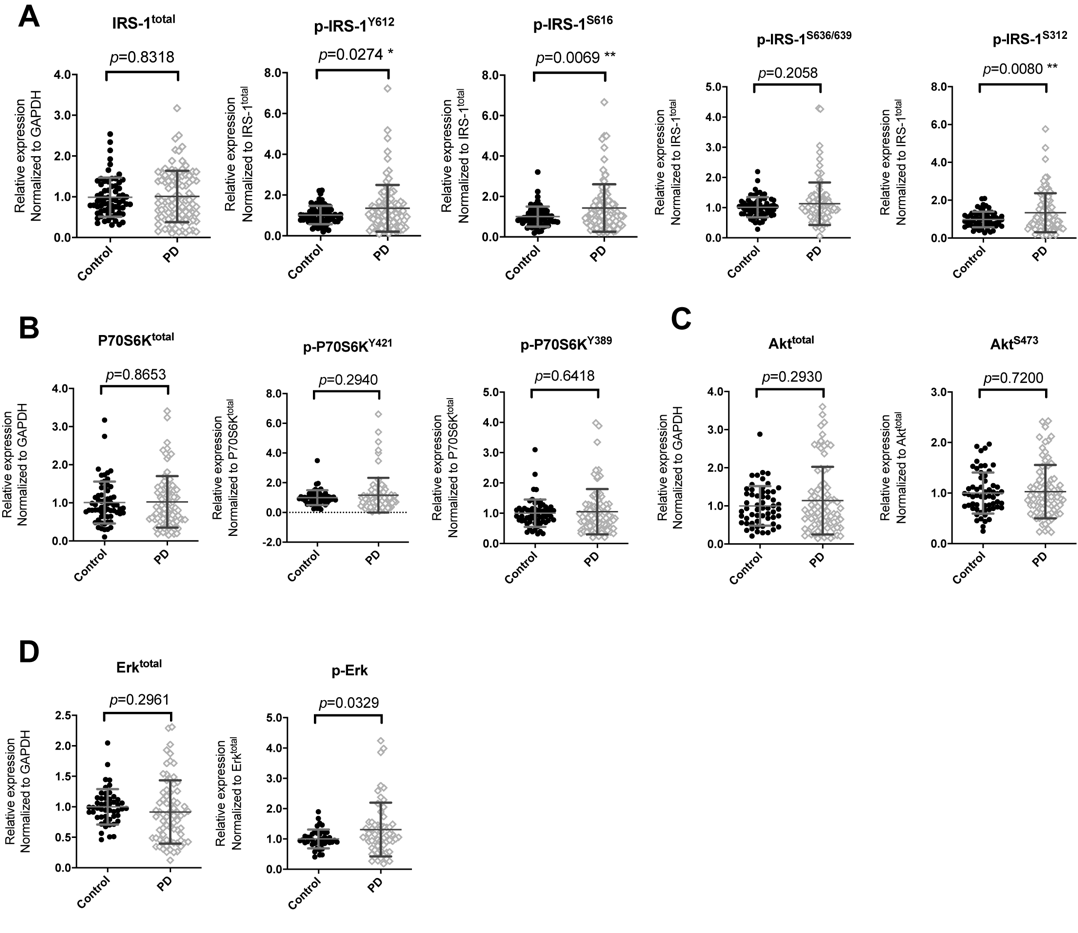
**

**Supplementary Table 1.** The correlation between level of p-IRS-1 S312 and S616 in blood NDEVs with individual item of motor symptoms (assessed by UPDRS-III) of PD in PD patients. *, *p*<0.05

|  | p-IRS-1 S312 | | p-IRS-1 S616 | |
| --- | --- | --- | --- | --- |
|  | Correlation coefficient | *p* value | Correlation coefficient | *p* value |
| Speech | -0.059 | 0.576 | 0.050 | 0.636 |
| Facial Expression | 0.137 | 0.194 | 0.060 | 0.572 |
| Rest tremor | 0.213 | 0.043* | 0.109 | 0.302 |
| Action tremor | 0.277 | 0.008* | 0.146 | 0.166 |
| Rigidity | -0.018 | 0.865 | 0.087 | 0.409 |
| Finger tapping | -0.122 | 0.251 | -0.101 | 0.340 |
| Hand grasping | -0.155 | 0.142 | -0.106 | 0.315 |
| Rapid alternative movement of forearm | -0.068 | 0.525 | -0.038 | 0.719 |
| Foot tapping | -0.077 | 0.466 | -0.102 | 0.331 |
| Arising from chair | 0.151 | 0.154 | 0.180 | 0.086 |
| Posture | 0.058 | 0.588 | 0.039 | 0.715 |
| Gait | -0.127 | 0.231 | -0.079 | 0.457 |
| Posture instability | 0.027 | 0.799 | -0.007 | 0.948 |
| General appearance | -0.068 | 0.524 | 0.014 | 0.895 |

**Supplementary Table 2** The correlation between the total IRS-1, each phosphorylated IRS-1, and the downstream protein total/phosphorylated from level in the neural derived exosomes in all PD patients.

Data was presented as the correlation coefficient (*r*). *, *p*<0.05, **, *p*<0.01, ***, *p*<0.001

|  | IRS-1 | p-IRS-1 Y612 | p-IRS-1 S616 | p-IRS-1 S636  /639 | p-IRS-1 S312 | P70S6K | P70S6K  T421 | P79S6K  T389 | Akt total | pAkt S473 | Erk  total | pErk |
| --- | --- | --- | --- | --- | --- | --- | --- | --- | --- | --- | --- | --- |
| IRS-1 | 1.000 |  |  |  |  |  |  |  |  |  |  |  |
|  |  |  |  |  |  |  |  |  |  |  |  |  |
| p-IRS-1 Y612 | -.567^***^ | 1.000 |  |  |  |  |  |  |  |  |  |  |
|  |  |  |  |  |  |  |  |  |  |  |  |  |
| p-IRS-1 S616 | -.554^***^ | .565^***^ | 1.000 |  |  |  |  |  |  |  |  |  |
|  |  |  |  |  |  |  |  |  |  |  |  |  |
| p-IRS-1 S636  /639 | -.388^***^ | .412^***^ | .311^**^ | 1.000 |  |  |  |  |  |  |  |  |
| p-IRS-1 S312 | -.305^**^ | .416^***^ | .491^**^ | .114 | 1.000 |  |  |  |  |  |  |  |
| P70S6K | .174 | .065 | .106 | .115 | -.059 | 1.000 |  |  |  |  |  |  |
| P70S6K  T421 | -.050 | -.300^**^ | .049 | -.175 | .028 | -.530^***^ | 1.000 |  |  |  |  |  |
| P79S6K  T389 | -.188 | -.066 | .146 | -.098 | .058 | -.513^***^ | .777^***^ | 1.000 |  |  |  |  |
| Akt total | -.115 | .108 | .242^*^ | .313^**^ | -.082 | .532^**^ | -.143 | -.072 | 1.000 |  |  |  |
| pAkt  S473 | .099 | .032 | -.081 | -.093 | .138 | -.185 | .305^*^ | .257^*^ | -.568^***^ | 1.000 |  |  |
| Erk  total | -.101 | .148 | .233 | .299^*^ | .230 | .253^*^ | .113 | -.034 | .396^**^ | -.069 | 1.000 |  |
| pErk | .201 | .040 | -.122 | -.262 | -.042 | -.149 | -.141 | -.072 | -.432^**^ | .193 | -.788^***^ | 1.000 |

**Supplementary Table 3.** The correlation between IRS-1 substrates in blood NDEVs with the severity of motor symptoms in PD patients. Data was presented as the correlation coefficient (γ) and none of the γ achieved the p value <0.05.

|  | P70S6K | pP70S6K  T421 | pP70S6K  T389 | Akt | pAkt  S473 | Erk | pErk |
| --- | --- | --- | --- | --- | --- | --- | --- |
| UPDRS-III | 0.026 | -0.041 | -0.059 | 0.071 | 0.116 | 0.077 | 0.075 |
| Tremor | -0.152 | -0.117 | -0.062 | -0.067 | 0.105 | 0.110 | 0.080 |
| Akinetic  rigidity | 0.151 | -0.074 | -0.166 | 0.089 | 0.054 | 0.097 | 0.177 |
| PIGD | 0.071 | 0.017 | 0.006 | 0.119 | 0.133 | 0.037 | 0.014 |
